# Supplementary material for: Assessing Genetic Variation in Wild and Domesticated Pikeperch Populations: Implications for Conservation and Fish Farming
Source: Animals (Basel). 2022 May 4;12(9):1178. doi: 10.3390/ani12091178 (PMC9102197; doi:10.3390/ani12091178)

Supplementary material

Table S1. Characteristics of the 11 microsatellite loci used in *Sander lucioperca* in this study. Optimized loci were grouped into two multiplexes. For each locus, we report the accession number in NCBI, the fluorescent dye we used in the automatic sequencer and the repeat motif (sequence). Size range, the number of alleles (Na) and the observed and expected heterozygosity (Ho and He, respectively) are provided for the species first described (Pfla for *Perca flavescens* [24], Svi for *Stizostedion vitreum* [25] and Za for *Zingel asper* [26]) and for pikeperch in this study. (‡) Locus PflaL3 was excluded from downstream analysis due to significant probability of allelic dropout. (*) Mean over populations

					**In species first described**				**In pikeperch**			
	**Locus**	**Acc. Number**	**Dye**	**Repeat motif**	**Size Range**	**Na**	**Ho**	**He**	**Size Range**	**Na**	**Ho***	**He***
**Multiplex1**	Svi18	G36964	6-FAM	$(AC)_{11}$	118–124	4	0.72	0.67	127-177	19	0.72	0.64
	PflaL3‡	AF211828	6-FAM	$(TG)_{18}$	130-186	6	0.58	0.75	96-114	9	0.45	0.51
	Za138	HM622317	Atto-565	$(AC)_{8}$	111-120	6	0.59	0.50	135-175	20	0.56	0.54
	Za199	HM622334	6-FAM	$(TCT)_{13}$	183-201	7	0.64	0.63	194-230	16	0.60	0.57
**Multiplex2**	Za038	HM622298	6-FAM	$(AC)_{11}$	102-110	4	0.66	0.50	110-140	13	0.67	0.62
	Svi4	G36961	Atto-550	$(AC)_{16}$	102–118	6	0.70	0.72	110-158	23	0.71	0.63
	Za024	HM622294	HEX	$(AC)_{7}$	104–106	2	0.22	0.20	118-146	14	0.44	0.45
	Za237	HM622342	6-FAM	$(CA)_{10}$	136-142	3	0.22	0.21	162-202	13	0.45	0.42
	Za144	HM622319	Atto-550	$(AC)_{8}$	186-190	3	0.59	0.59	201-239	17	0.66	0.62
	PflaL9	AF211834	HEX	$(TG)_{24}$	214–300	11	0.67	0.71	183-213	8	0.44	0.49
	Za207	HM622337	Atto-565	$(GT)_{13}$	174-195	5	0.50	0.42	216-240	11	0.63	0.57

Table S2. *Cyt b* haplotypes used in the phylogeographic analysis

Origin				Hungary	Netherlands	Czech	Netherlands	Hungary	Denmark	Germany	Finland	Finland	France	Germany	Netherlands		Wild								Data from Genbank										
**Haplotype**	**Samples sequences**	**Reference**	**Acc. Number**	**Hungary-2**	**Denmark-1**	**Denmark-2**	**Denmark-3**	**Denmark-4**	**Denmark-5**	**Germany**	**Finland-1**	**Finland-2**	**France-1**	**Belgium-1**	**Belgium-2**	**Sweden**	**Hungary-1**	**Tunisia**	**France-2**	**Czech**	**Poland-1**	**Poland-2**	**Finland-3**	**Finland-4**	**Germany**	**Hungary**	**Poland**	**France**	**Czech**	**Finland**	**Serbia**	**Azerbaijan**	**Iran**	**Russia**	Original Haplotype
**H01**	**5**	THIS STUDY	**ON245529-ON245533**	2													3																		H01
	**1**	[3]	**KC960521.1**																						1										B2
	**1**	[2]	**KC819826.1**																												1^^				Slucb4
**H02**	**4**	THIS STUDY	**ON245534-ON245537**							4																									H02
**H03**	**56**	THIS STUDY	**ON245538-ON245593**	8				9		4				10			9	14			1	1													H03
	**1**	[46]	**KM410087.1**																							1*									
	**1**	[3]	**KC960520.1**																						1										B1
	**1**	[3]	**KC960519.1**																						1										B
	**1**	[2]	**KC819825.1**																								1							1	Slucb3
	**1**	[11]	**JX025365.1**																									1**							B1
	**1**	[11]	**JX025364.1**																						1***										B
	**1**	[44]	**FJ788397.1**																										1						
**H04**	**16**		**ON245594-ON245609**						10						1				1		1	3													H04
	**1**	[3]	**KC960517.1**																						1										A1
	**1**	[11]	**JX025363.1**																									1^							A1
**H05**	**131**	THIS STUDY	**ON245610-ON245740**		10	11	10	1		1	11	10	10	1	9	9			12	10	4		11	11											H05
	**1**		**KP125333.1**																																
	**1**	[3]	**KC960516.1**																						1										A
	**1**	[2]	**KC819824.1**																													1		1	Slucb2
	**1**	[44]	**FJ788390.1**																										1						
	**1**	[45]	**GU936790.1**																											1					
	**1**		**GQ214533.1**																														1		
**H06**	**1**	[3]	**KC960518.1**																						1										A2
**H07**	**2**	[2]	**KC819823.1**																															2	Slucb1
**H08**	**1**		**AF546122.1**																																

^ Retenue de Hautefage, ^^Danube River, *Balaton Lake- Keszthely, ** Etang des Aulnes, *** Lake Mueggelsee

Table S3. Number of alleles per locus per population.

Population	Locus										
	PflaL3	Svi18	Za199	Za138	PflaL9	Svi4	Za024	Za038	Za144	Za207	Za237
**Hungary-2**	5	12	5	14	4	6	6	8	8	6	9
**Denmark-1**	3	3	2	3	2	4	2	3	3	2	2
**Denmark-2**	3	4	3	3	3	5	3	4	3	3	2
**Denmark-3**	2	4	1	2	1	5	2	4	4	3	2
**Denmark-4**	5	13	6	13	3	8	7	9	8	6	9
**Denmark-5**	2	4	3	2	4	5	2	3	3	3	2
**Germany**	2	4	4	7	5	6	6	4	9	7	5
**Finland-1**	3	4	4	4	3	5	4	4	4	4	1
**Finland-2**	2	3	3	2	5	4	3	3	1	2	2
**France-1**	5	7	7	6	4	5	4	4	8	6	3
**Belgium-1**	4	9	10	9	6	7	5	5	9	6	6
**Belgium-2**	5	5	7	7	5	5	3	3	4	4	4
**Sweden**	1	3	7	4	4	7	4	6	3	3	3
**Hungary-1**	4	6	5	9	3	7	7	6	8	4	7
**Tunisia**	3	5	6	4	2	3	1	3	6	4	3
**France-2**	5	6	5	5	4	6	3	4	6	4	3
**Czech Rep.**	3	5	4	6	3	5	3	4	3	3	2
**Poland-1**	5	6	7	4	4	4	4	5	5	4	3
**Poland-2**	3	4	7	4	4	3	4	4	4	5	3
**Finland-3**	4	5	8	5	4	6	5	4	3	4	4
**Finland-4**	5	5	5	6	5	7	5	4	4	3	3
**Total No. Alleles**	**9**	**19**	**16**	**20**	**8**	**23**	**14**	**13**	**17**	**11**	**13**
**Mean No. Alleles per Locus**	**3.5**	**5.6**	**5.2**	**5.7**	**3.7**	**5.4**	**4.0**	**4.5**	**5.0**	**4.1**	**3.7**

Table S4. Pairwise F_{st} values calculated for microsatellites with Arlequin (distance method: number of different alleles). All values are statistically significant (p<0.05). Grey background is highlighting the smallest values (<10%) while bold and underlined numbers the highest values (>40%).

	1	2	3	4	5	6	7	8	9	10	11	12	13	14	15	16	17	18	19	20
**2**	0.262																			
**3**	0.258	0.124																		
**4**	0.264	0.254	0.180																	
**5**	0.010	0.268	0.271	0.274																
**6**	0.342	0.303	0.346	0.412	0.339															
**7**	0.227	0.156	0.108	0.202	0.233	0.259														
**8**	0.326	0.368	0.394	**0.416**	0.320	0.239	0.323													
**9**	0.349	**0.450**	**0.472**	**0.484**	0.340	0.362	0.393	0.209												
**10**	0.215	0.147	0.184	0.221	0.214	0.239	0.159	0.298	0.343											
**11**	0.136	0.186	0.163	0.190	0.145	0.209	0.159	0.205	0.257	0.132										
**12**	0.257	0.292	0.302	0.279	0.264	0.151	0.248	0.219	0.238	0.245	0.192									
**13**	0.293	0.273	0.316	0.333	0.290	0.160	0.257	0.169	0.300	0.231	0.178	0.174								
**14**	0.087	0.275	0.256	0.282	0.106	0.374	0.234	0.359	0.381	0.213	0.167	0.288	0.333							
**15**	0.316	0.259	0.224	0.343	0.322	0.292	0.203	0.388	**0.475**	0.223	0.224	0.249	0.334	0.328						
**16**	0.190	0.105	0.105	0.213	0.204	0.242	0.116	0.305	0.364	0.046	0.118	0.252	0.235	0.175	0.209					
**17**	0.252	0.134	0.034	0.150	0.267	0.379	0.141	0.426	**0.480**	0.187	0.175	0.334	0.349	0.257	0.293	0.126				
**18**	0.240	0.203	0.254	0.281	0.252	0.153	0.194	0.229	0.329	0.201	0.161	0.163	0.143	0.261	0.302	0.186	0.278			
**19**	0.231	0.254	0.289	0.308	0.238	0.091	0.199	0.195	0.250	0.187	0.138	0.097	0.157	0.278	0.278	0.188	0.303	0.037		
**20**	0.284	0.327	0.349	0.353	0.282	0.183	0.291	0.059	0.150	0.258	0.165	0.150	0.134	0.317	0.359	0.263	0.380	0.161	0.116	
**21**	0.288	0.316	0.355	0.370	0.286	0.193	0.293	0.076	0.153	0.253	0.163	0.185	0.099	0.317	0.364	0.262	0.384	0.149	0.123	0.023

Table S5: Structure Harvester results.

K	Reps	Mean LnP(K)	Stdev LnP(K)	Ln'(K)	|Ln''(K)|	Delta K
**1**	10	-33412.49	0.06	—	—	—
**2**	10	-29233.30	0.69	4179.19	1894.25	2734.11
**3**	10	-26948.36	1.36	2284.94	1428.33	1047.18
**4**	10	-26091.75	137.92	856.61	294.48	2.14
**5**	10	-25529.62	388.76	562.13	373.36	0.96
**7**	10	-24369.64	328.18	971.21	613.60	1.87
**8**	10	-24012.03	61.00	357.61	251.47	4.12
**9**	10	-23905.89	83.39	106.14	31.33	0.38
**10**	10	-23768.42	161.81	137.47	167.02	1.03
**11**	10	-23797.97	211.78	-29.55	125.82	0.59
**12**	10	-23701.70	119.47	96.27	902.74	7.56
**13**	10	-24508.17	2524.81	-806.47	71.61	0.03
**14**	10	-25386.25	5261.80	-878.08	96.20	0.02
**15**	10	-26360.53	5912.52	-974.28	3670.72	0.62
**16**	10	-23664.09	25.48	2696.44	2749.36	107.90
**17**	10	-23717.01	33.60	-52.92	39.01	1.16
**18**	10	-23808.94	74.33	-91.93	115.67	1.56
**19**	10	-23785.20	24.08	23.74	111.57	4.63
**20**	10	-23873.03	104.08	-87.83	94.92	0.91
**21**	10	-23865.94	30.80	7.09	124.77	4.05
**22**	10	-23983.62	98.60	-117.68	143.86	1.46
**23**	10	-23957.44	30.07	26.18	57.62	1.92
**24**	10	-23988.88	22.21	-31.44	47.07	2.12
**25**	10	-24067.39	76.35	-78.51	—	—

Table S6. AMOVA results based on mtDNA polymorphism.

	Scenario 1 - "K=2"	Scenario 2 - "K=3"	Scenario 3 - "DAPC"	Scenario 4 - "Φ_{ST}"
Group 1	Hungary-1,2, Denmark-1,2,3,4, Germany, France-1,2, Belgium-1, Tunisia, Czech Rep.	Hungary-1,2, Denmark-4, Belgium-1	Hungary-1,2, Denmark-4,	Hungary-1,2, Denmark-4, Germany, Belgium-1, Tunisia
Group 2	Denmark-5, Finland-1,2,3,4, Belgium-2, Sweden, Poland-1,2	Denmark-1,2,3, France-1,2, Germany, Tunisia, Czech Rep.	Finland-1,2,3,4, Sweden	Denmark-1,2,3,5, Czech Rep., Finland-1,2,3,4, Sweden, Belgium-2, France-1,2, Poland-1,2
Group 3		Denmark-5, Finland-1,2,3,4, Belgium-2, Sweden, Poland-1,2	Denmark-1,2,3,5, Germany, Czech Rep., France-1,2, Tunisia, Belgium-1,2, Poland-1,2	
Variation %				
Among groups	28.99	48.02	36.55	85.59
Among populations within groups	55.12	36.30	47.70	4.56
Within populations	15.89	15.68	15.75	9.85
Fixation Indices				
Φ_{ST}	0.8411	0.8432	0.8426	0.9014
p-value	<0.0001	<0.0001	<0.0001	<0.0001
Φ_{SC}	0.7762	0.6983	0.7518	0.3161
p-value	<0.0001	<0.0001	<0.0001	<0.0001
Φ_{CT}	0.2899	0.4802	0.3655	0.8559
p-value	0.0006	<0.0001	0.0004	<0.0001

Figure S1. Graphical representation of mean number of alleles per locus (Na), unbiased expected heterozygosity (uH$_E$), and allelic richness (A$_r$) in wild and domesticated populations, ± SEM

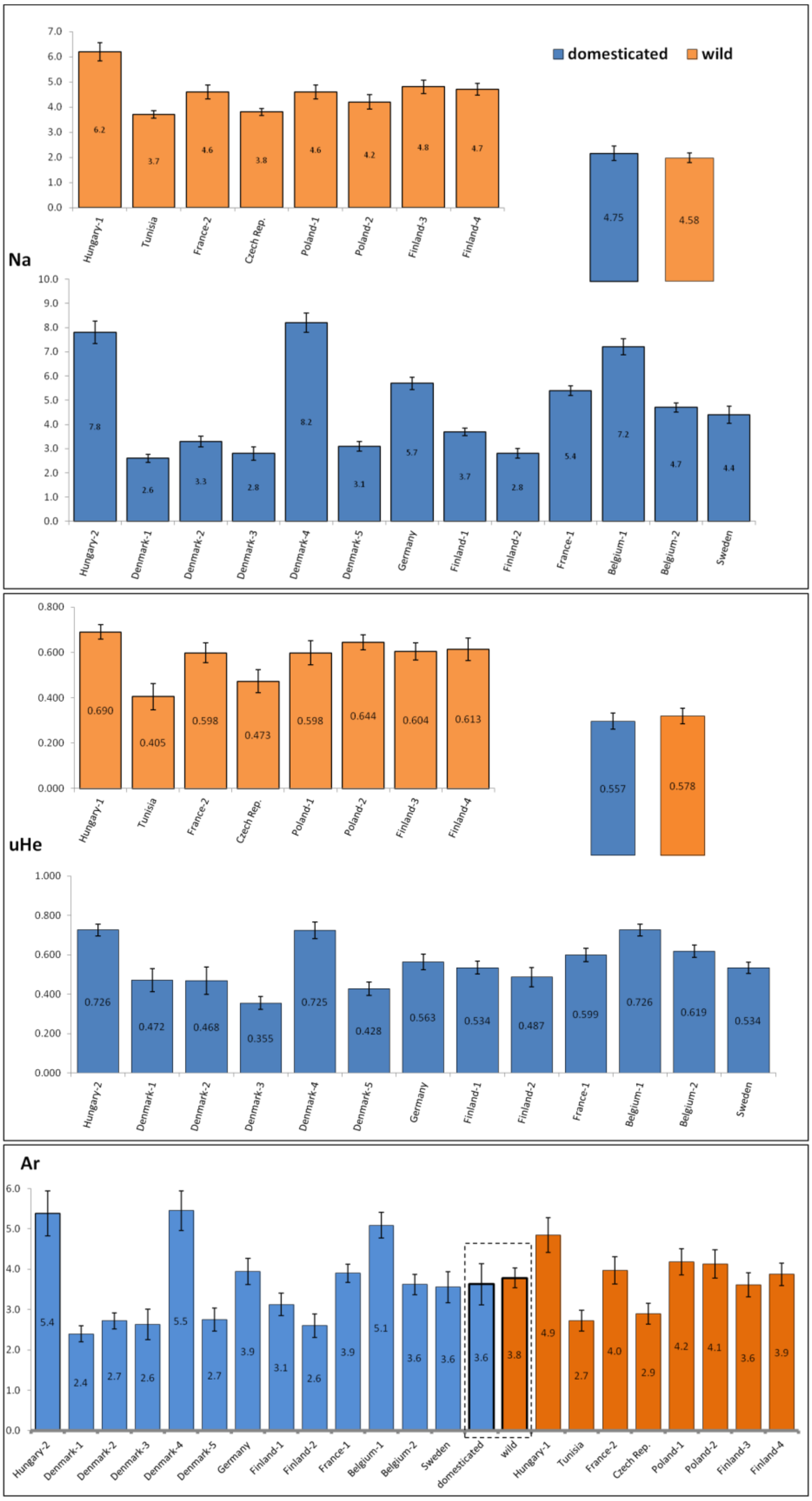

Figure S2. Matrix of pairwise Φ_{ST} values

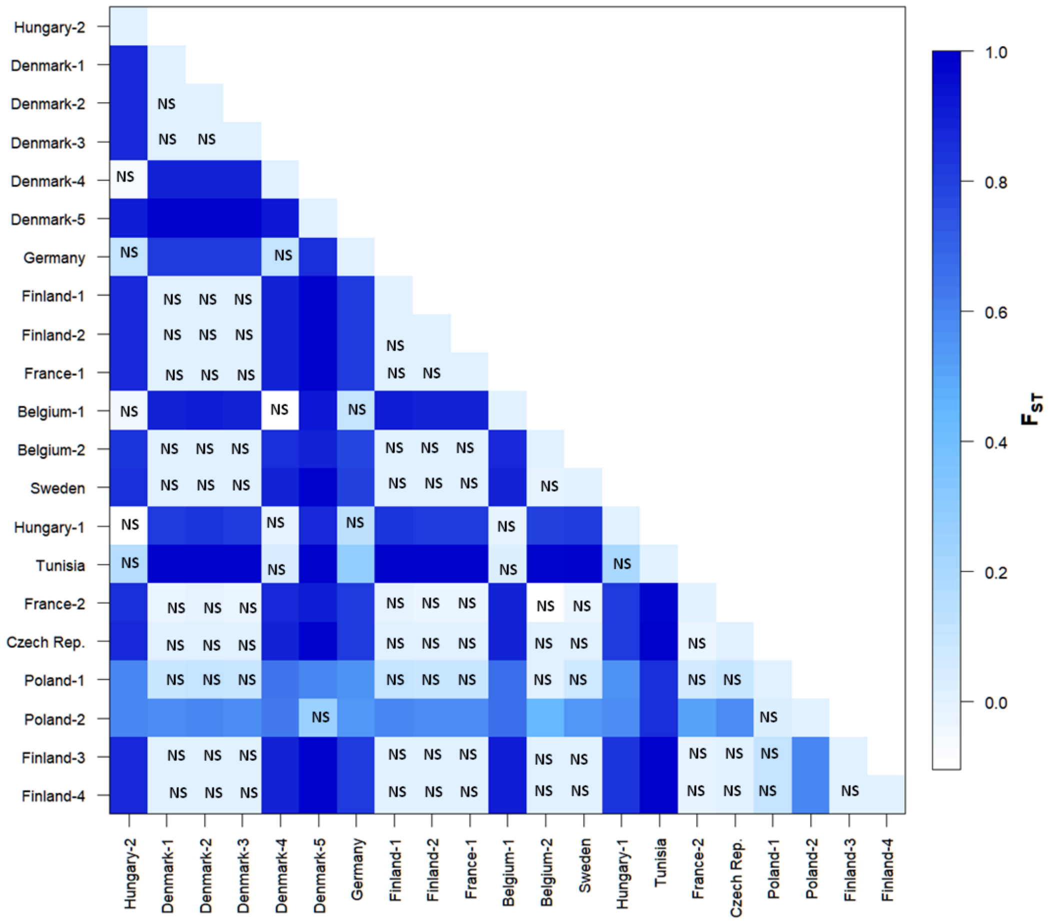

Supplement: Supplementary file 1 [file animals-12-01178-s001.zip › animals-1641397-supplementary.pdf]
